# Relevant Aspects of Titanium and Zirconia Dental Implants for Their Fatigue and Osseointegration Behaviors

**DOI:** 10.3390/ma15114036

**Published:** 2022-06-06

**Authors:** Javier Aragoneses, Nansi Lopez Valverde, Manuel Fernandez-Dominguez, Jesús Mena-Alvarez, Cinthia Rodriguez, Javier Gil, Juan Manuel Aragoneses

**Affiliations:** 1 Department of Medicine and Medical Specialties, Faculty of Medicine and Health Sciences, Universidad Alcalá de Henares, 28871 Madrid, Spain; jaragoneses@uax.es (J.A.); nlopez@uah.es (N.L.V.); 2 Department of Translational Medicine, CEU San Pablo University, 28660 Madrid, Spain; clinferfu@yahoo.es; 3 Faculty of Dentistry, Universidad Alfonso X el Sabio, C. de Emilio Muñoz, 13, 28691 Madrid, Spain; jmena@uax.es (J.M.-A.); jaraglam@uax.es (J.M.A.); 4 Department of Dentistry, Universidad Federico Henriquez y Carvajal, Santo Domingo 10106, Dominican Republic; crodriguez@uhercar.do; 5 Bioengineering Institute of Technology, Facultad de Medicina y Ciencias de la Salud, Universitat Internacional de Catalunya, 08195 Sant Cugat del Vallés, Spain

**Keywords:** osseointegration, titanium, zircona, dental implants, bone index contact, histology

## Abstract

Osseointegration capacity and good mechanical behavior are key to the success of the dental implant. In many investigations, comparisons of properties are made using different dental implant designs and therefore the results can be influenced by the macrodesign of the dental implant. In this work, studies were carried out with the same dental implant model using different roughness and different materials—commercially pure titanium (grade 4) and zirconia. For this purpose, 80 smooth passivated titanium (Ti), 80 smooth zirconia (ZrO_2_), and 80 rough passivated titanium (Ti-R) dental implants were used. The samples were characterized by their roughness, wettability, surface energy, residual stresses, and fatigue behavior. The implants were implanted in minipigs for 4 and 12 weeks. The animals were sacrificed, and histological studies were carried out to determine the osseointegration parameters for each of the implantation times. Ti and ZrO_2_ dental implants have very similar wettability and surface energy properties. However, the roughness causes a decrease in the hydrophilic character and a decrease of the total surface energy and especially the dispersive component, while the polar component is higher. Due to the compressive residual stresses of alumina sandblasting, the rough dental implant has the best fatigue behavior, followed by Ti and due to the lack of toughness and rapid crack propagation the ZrO_2_ implants have the worst fatigue behavior. The bone index contact (BIC) values for 4 weeks were around 25% for Ti, 32% for ZrO_2_, and 45% for Ti-R. After 12 weeks the Ti dental implants increased to 42%, for Ti, 43% for ZrO_2_, and an important increase to 76% was observed for Ti-R implants. In vivo results showed that the key factor that improves osseointegration is roughness. There was no significant difference between ZrO_2_ and Ti implants without sandblasting.

## 1. Introduction

Titanium and Ti6Al4V dental implants have been successful in replacing teeth due to their osseointegration capability, good mechanical properties—especially in fatigue due to cyclic chewing loads—their high corrosion resistance, and their aesthetic ability in the mouth. Currently, the most commonly used dental implants are made of commercially pure titanium. Titanium has four grades of titanium depending on the degree of impurities, with grade 3 and 4 implants being the most commonly used due to their good mechanical properties [1,2,3,4,5,6]. In some cases, some companies work with the titanium alloy Ti6Al4V whose mechanical properties are higher than titanium, but which has poorer corrosion resistance and cytocompatibility properties [7,8,9,10,11]. Titanium produces a spontaneous film of titanium oxide, which makes the material more resistant to corrosion and reduces titanium ion release [6,7,8]. This titanium oxide layer can be made larger by applying passivation agents to the titanium, such as citric acid, nitric acid, or Piranha reagent [9,10,11,12,13,14,15,16].

The development of bioceramics for dental implantology is currently very important. Among the different bioceramics, the yttria-stabilized tetragonal zirconia (Y-TZP) is not only gaining interest as prosthesis but also as a dental implant. The Y-TZP ceramics can be found as monolithic—implant and abutment in only one piece—which require extra efforts to optimize their structure, or can be composed of two separate articulated pieces, having the implant and the abutment as two separate pieces [17,18,19,20,21,22,23,24,25,26,27].

This bioceramic presents a very important increase in toughness due to the solid-state transformation induced by stress. When the external stresses provoke a growing crevice in the material, the local stresses at the crack tip induce transformation from tetragonal to monoclinic phase around the crack tip. This tetragonal-to-monoclinic transformation causes a volume growth of around 4%. The volume growth around the crack tip induces compressive stresses that tend to close the crack, increasing the fracture toughness of the material [16,17,18]. Bioceramics present some advantages such as avoiding corrosion, improving the wear resistance as well, and presenting good aesthetic properties. However, the ceramics are brittle and when used as ceramic implants, they present lower toughness than the c.p. Titanium [19,20,21,22,23,24]. A disadvantage of zirconia dental implants is that the roughness suitable for osteoblastic activity cannot be obtained as the ceramic does not allow impacts from abrasive materials due to its brittleness.

Both titanium and zirconia dental implants present a good corrosion behavior in physiological media at 37 °C due to their bioinert character under normal working conditions. There was some controversy about the degradation of zirconia due to leaching of the yttria which causes volume increases in the material and could lead to breakage and premature failure. However, this degradation can only occur in the femoral balls of hip prostheses due to the continuous wear forces that can cause such degradation. In zirconia dental implants the degeneration is only in the first few nanometers of the surface and does not affect the reliability and structural integrity of the dental implant [20,24,25,26,27]. The biocompatibility of both titanium and zirconia are excellent at all pH ranges, no variation in properties has been observed due to pH changes in inflammation processes where the pH becomes more acidic, nor at the more basic pHs to which they may be subjected [9,10,25]. New techniques for the production of implants with calcium phosphate coatings have been developed to obtain bioactive surfaces with very good results [26,27].

In the literature there are many published works on dental implant designs [28,29,30,31,32,33,34,35,36,37,38,39,40] and on the influence of roughness on physico-chemical and fatigue properties as well as on osseointegration of dental implants [40,41,42,43,44,45,46,47]. However, there are not many studies on the influence of the nature of the implant material using the same design and topography. This work aimed to determine the influence of the nature of the material and the role of the roughness using the same design of the implants. In this contribution we were able to demonstrate the osseointegration capability of commercially pure titanium and zirconia with the same roughness and design of the dental implant, which has not been possible until now. Furthermore, we were able to confirm the fundamental role of roughness in the osseointegration and mechanical properties of titanium dental implants based on the compressive residual stress generated in sand blasting treatment.

## 2. Materials and Methods

### 2.1. Dental Implants

**Figure 1:**
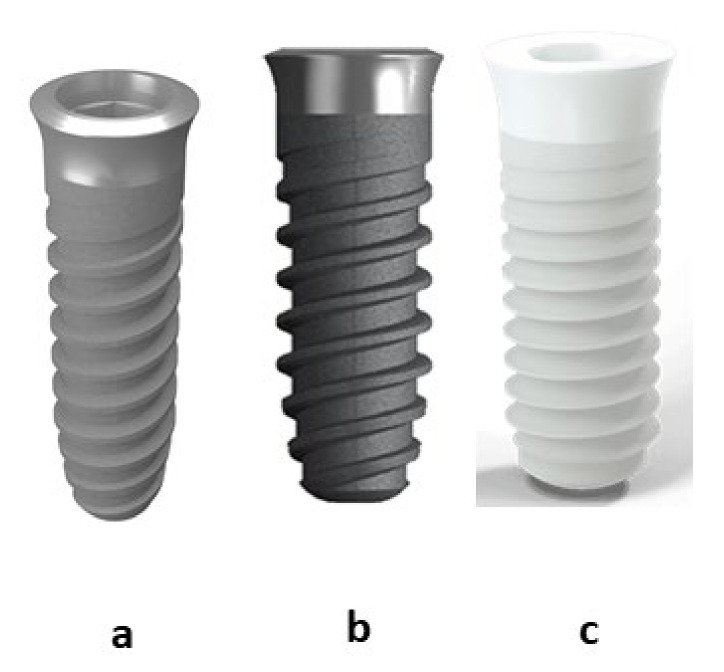
Dental implants used in this study. (**a**) Titanium passivated (Ti). (**b**) Titanium rough and passivated (Ti R). (**c**) Zircona-YZP (ZrO_2_).

Three types of implants with the same macroscopic and design characteristics were studied (Figure 1). The titanium dental implants are of Essential internal connection (Klockner Dental Implants, Escaldes Engordany, Andorra) one was sand blasted with alliumine and the other was machined as obtained from the machine. The zirconia dental implants were obtained with the same geometry and identical design but by sintering zirconia powder with yttria. The zirconia dental implants are not sandblasted as roughness would not be achieved due to the hardness of zirconia. Sintering molds cannot be roughened to facilitate the removal of dental implants.

- Commercially pure titanium cp-Ti (grade 4) (Klockner Dental Implants, Escaldes Engordany, Andorra) smooth with passivated treatment (citric acid 20% for 15 s) (Ti).
- Commercially pure titanium cp-Ti (grade 4) sand blasted with alumina (350 μm to 500 μm) projected at 2.5 bars and 200 mm of distance (implant-gun). After the sand blasted treatment, the samples were passivated (citric acid 20% for 15 s) (Ti-R)
- Zirconia-2.5 Y-TZP. (ZrO_2_)

Different topographies were characterized by scanning electron microcopy (SEM) (Jeol 6700, Tokyo, Japan) using an acceleration potential of 20 KeV. The chemical composition of the metallic dental implants was determined by EDS microanalysis accoupled in the SEM and for the zircona implant an X-Ray diffractometer (Siemens, Berlin, Germany) was used in order to obtain the oxides percentages.

### 2.2. Roughness

For the determination of roughness, an Olympus LEXT OLS3100 (Tokyo, Japan) confocal microscope was used for the different study groups. Three samples from each group were used and 3 measurements per sample were taken at ×1000 magnification. The parameters R_a_ and R_z_ were determined. Ra corresponds to the arithmetic mean of the absolute values of the deviations of the profiles of a given length of the sample. Rz corresponds to the sum of the maximum peak height and the maximum valley depth within the sampling length.

### 2.3. Contact Angle and Surface Free Energy

Wettability and surface energy of samples were measured using a contact angle system OCA15plus (Dataphysics Instrument Company, Filderstadt, Germany) and results were analyzed with SCA20 software (Dataphysics Instrument Company, Filderstadt, Germany). Contact angle (CA) and surface free energy (SFE) were determined by using the traditional sessile drop measurement method in the static mode. The aforementioned process allows the measurement of the angle θ formed between the water drop and the surface. The greater the contact angle, the lower the wettability and vice versa. For angles less than 10° the surface is considered superhydrophilic, for angles between 10° and 90° hydrophilic, and for angles greater than 90° hydrophobic. A droplet generation system equipped with a 500 μL Hamilton syringe with micrometric displacement control was used to control the volume (3 μL) and to deposit the droplet.

Two different reference liquids were used to calculate the surface energy, measuring the contact angle values using ultra-distilled Milie-Q grade (Millipore Milie-Q Merck Millipore Corp., Darmstadt, Germany) as a polar liquid and di-iodomethane (Sigma Aldrich, St. Louis, MO, USA) as a non-polar liquid, respectively. The contact angle measurements of di-iodomethane were obtained following the same procedure as for water.

The surface energy was calculated using (Equation (1)) the Owens and Wendt equation:

(1) $$\gamma_{L}\cdot (1+\cos\theta )=2\cdot ({\left(\gamma_{L}^{d}\cdot \gamma_{S}^{d}\right)}^{1/2}+{\left(\gamma_{L}^{p}\cdot \gamma_{S}^{p}\right)}^{1/2})$$

where γ_*d*_ and γ_*p*_ represent the dispersive and polar components respectively of the liquid used and is the angle between the solid and the liquid. The total surface energy of a surface equals the sum of its dispersive and polar components.

### 2.4. Residual Stresses

Residual stresses were measured with a diffractometer incorporating a Bragg–Bentano configuration (D500, Siemens, Germany). The measurements were done for the family of planes (213), which diffracts at 2θ = 139.5°. The elastic constants of Ti at the direction of this family of planes are EC = (E/1 + υ)_(213)_ = 90.3 (1,4) GPa. Eleven ψ angles, 0° and five positive- and five negative-angles were evaluated. The position of the peaks was adjusted with a pseudo-Voigt function using appropriate software (WinplotR, free access on-line), and then converted to interplanar distances using Bragg’s equation. The dψ vs. sen2ψ graphs and the calculation of the slope of the linear regression (A) were conducted with appropriate software (Origin, Microcal, Northampton, MA, USA). The residual stress is: σ = EC ∗ (1/d_0_) ∗ A; where d_0_ is the interplanar distance for ψ = 0°.

### 2.5. Fatigue Behavior

The fatigue behavior and the fatigue limit of the prototype were set using Wöhler’s curves (stress-number of cycles) that describe the relation between the amplitude of the cyclical tensions and the number of cycles up to break. During the test, the implant-abutment system was subjected to both cyclical compressive and lateral forces, without any lateral constraint. Fifty implants for each type of implant were tested.

The tests were performed in simulated body fluid at 37 °C with the servo-hydraulic testing machine MTS Bionix 858, which is specially designed to test biomaterials. This machine was equipped with a load cell MTS of 25 KN and controlled by means of a PC equipped with the software TESTAR II^®^ (Tokyo, Japan).

**Figure 2:**
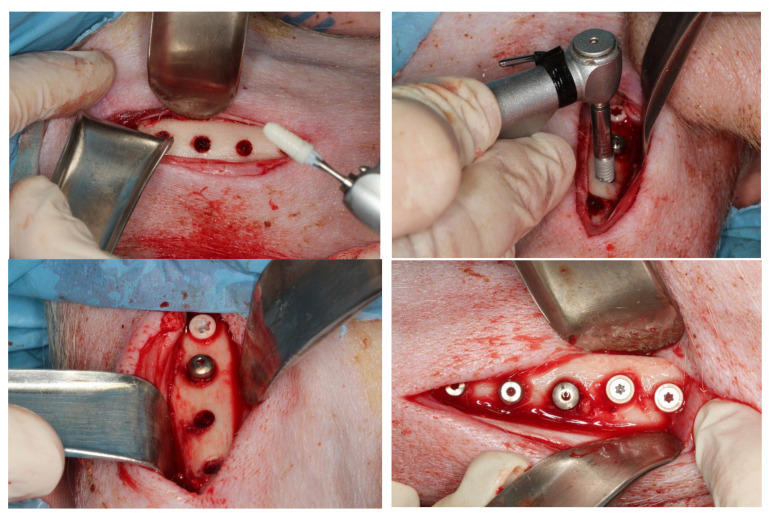
Images of the surgical procedure for implant placement.

The tests were performed following the guidelines previously published by the FDA for the Class II Special Controls Guidance Documents: Roots-form Endosseous Dental Implants and Endosseous Dental Implants Abutments and the ISO 14801:2007. The tested implants supported an abutment that was in line with the axis of the implant. The testing setup clamped the implant so that the implant’s long axis made a 30° angle with the loading direction of the testing machine and, consequently a flexural load was applied (Figure 2). The implants were fixed with a 30° inclination from the *z*-axis of the traction-compression machine. A 30° angle to the *z*-axis of the tensile-compression machine is recommended by the standards of the FDA as the most unfavorable position. Moreover, the implant was placed 3mm below the anticipated crestal bone level, simulating 3 mm of bone resorption.

To start with, five resistance tests were conducted at the selected inclination, to determine the yield strength of the material and the ultimate flexion strength. The different percentages of yield strength that were obtained from these results, ranging from 60 to 90%, were used later to perform fatigue tests to obtain the number of cycles until fracture.

The aim was to find the level of stress at which the sample supports five million cycles, and which can be considered the fatigue limit. Seven of the tests that were carried out to determine the level of stress analyzed the fatigue limit whilst three tests analyzed the rest of the tested stresses. The implants were loaded with a sinusoidal function of fatigue at a frequency of 15 Hz and the relationship between maximum and minimum applied stress was 10%. The tests were performed at room temperature. The obtained data were represented as the number of cycles to failure as a function of applied stress.

### 2.6. In Vivo Study

All animal procedures in this study were performed in compliance with the Guide for Care and Use of Laboratory Animals (National Research Council, in: Guide for the Care and Use of Laboratory Animals, National Academy Press, Washington, DC, USA, 1996, pp. 41–194) and the European Community Guidelines for the protection of animals used for scientific purposes (Directive 2010/63/EU of the European Parliament and of the Council of 22 September 2010 on the Protection of Animals Used for Scientific Purposes), and under the permission of the National Committee on Human and Animal Research (ref# UAB-CEAAH 2016).

A total of 90 implants (30 for each type of implant) were inserted in the tibiae of the 9 minipigs. Clinical follow-up was carried out and the animals were subsequently sacrificed after 4 and 12 weeks, depending on the group to which they belonged. To establish standard conditions for the histomorphometric analysis, the implants were placed in the tibiae of the animals as this bone shows a constant geometric pattern over almost its entire length. The values obtained from the histomorphometric analysis therefore depended only on the implant and not on the bone characteristics at the implantation site.

### 2.7. Surgical Intervention

The animals remained on a solid fast for 18 h prior to surgery and on a liquid fast for 6 h prior to surgery. Due to this prolonged fasting, oral solutions containing electrolytes and glucose were administered. The animals were intubated endotracheally.

Surgical premedication was performed intramuscularly in the neck of the animal with atropine sulphate (1 mg sulfuricum; Aguettant, Lyon, France), carazolol (Suacron^®^, Barcelona, Spain), and azaperone (Stressnil ^®^Norvet, Lugo, Spain) (2 mg/kg). 20 min later, an intramuscular combination of ketamine (10 mg/kg, Pfizer, New York, NY, USA) and midazolan (3 mL, Roche, Basel, Switzerland) was used for anesthetic induction. Anesthesia during surgery was balanced with oxygen (Air liquide Medicinal, Madrid, Spain), nitrous oxide (Air liquide Medicinal, Madrid, Spain), and isofluorane (Inibsa, Barcelona, Spain) (1.5%).

Surgeries were performed under sterile conditions in the veterinary operating theatre. The lower extremities of the animals were shaved, washed, and decontaminated with povidone–iodine (Figure 2). For implantation, a longitudinal medial skin and fascial incision was first made in the lower limb of the animal extending from the proximal epiphysis to the distal epiphysis with a scalpel handle (BXB-5 Bonfanti & Gris) and a number 15 scalpel blade (Henry Schein). This incision is intended to avoid damage to anatomical structures such as the saphenous vascular nerve bundle. With the help of a Molt periostotome (Molt BRM-24 Bonfanti & Gris), the aim is to uncover the medial aspect of the tibial diaphysis as well as part of the tibial metaphysis.

After debonding, the osteotomy was performed with the drilling sequence recommended by the manufacturer (for GMI^®^ Frontier implants, Lleida, Spain) and irrigation with physiological saline solution. (Omnirigator sterile irrigation set Proclinic) The implants were then inserted (4/5 implants for each tibia) with a motor (W&H implantmed) and surgical contra-angle handpiece (WS-92 LG), recording the insertion torque of each implant. All implants in both groups had the same dimensions: Diameter 4 mm and length 10 mm.

As postoperative medication, 1.5 g of intramuscular amoxicillin (Clamoxyl^®^, Pfizer, Madrid, Spain) and 0.1 mg/kg intravenous butorphanol as analgesic (Torbugesic^®^, Laboratorios Fort Dodge, Gerona, Spain) were administered as antibiotherapy. For sacrifice, the animals were given an overdose of intracardiac sodium pentobarbital. (Eutha 77^®^, Pfizer, New York, NY, USA). Subsequently, the tibiae were cut, separated from the body, shaved and all soft tissue was removed, preserving only the bone.

### 2.8. Histomorphometric/Histological Analysis

This was carried out after sacrifice 4 or 12 weeks after implant insertion. In the histometric analysis of the samples, 5 measurements were analyzed, the percentage of bone contact implant bone contact or BIC and cortical implant bone contact or BICc, the peri-implant and inter-implant bone density, and the percentage of new bone or BV/TV of new bone. Two studies by Nkenke and Kuchler were used as the basis for the analysis of the measurements [48,49,50]. The BIC was defined as the amount of perimeter surface of the implant in direct contact with the bone tissue. This data was expressed as a percentage, so the amount of surface in direct contact with the bone was divided by the total perimeter of the implant (with the understanding of total perimeter from the neck of the implant to half of its length, everything being included in the marked box). The BICc is like the BIC but includes only the portions of the implant that cross the cortical bone section. The bone density inside the threads was defined as the surface area of bone that had grown inside the threads in relation to the total available interthread space (BAI/TA).

Areas analyzed histomorphometrically were as follows: Both in test and control implants; implant neck, neck-first spiral, middle third of the implant, middle third-self-tapping area, self-tapping area, most apical part of the implant.

### 2.9. Specimen Preparation

Specimens were processed using methacrylate embedding techniques described by Schlegel, Donath et al. [44].

− Fixation: Bone blocks were placed in 10% formalin for 2 weeks.
− Dehydration: With alcohols at different concentrations under constant agitation:

70% alcohol for 3 days
80% alcohol for 3 days
96% alcohol for 3 days
99.8% alcohol for 3 days

Plastic infiltration was performed by mixing glycolmethacrylate (Technovit 7200^®^, VLC-Heraus Kulzer GMBH, Werheim, Germany) and 1% benzoyl peroxide (BPO^®^: Heraus Kulzer GMBH, Werheim, Germany) with ethyl alcohol at different concentrations, ending with two infiltrations of pure glycolmethacrylate, under constant agitation (Exakt 510), according to the following procedure:

Technovit 7200^®^ + BPO: alcohol (30:70) for three days.
Technovit 7200^®^ + BPO: alcohol (50:50) for three days.
Technovit 7200^®^ + BPO: alcohol (70:30) for three days.
Technovit 7200^®^ + BPO (100) for three days.
Technovit 7200^®^ + BPO (100) for three days under vacuum.

For the inclusion, the tissue samples were placed in polyethylene molds which were subsequently filled with resin (Technovit 7200^®^) under vacuum (Exakt 530 and 520). The polymerization process was carried out in 3 phases: 1. Low intensity light was used for a period of 4 h. The molds were kept at a temperature of 40 °C. 2. For 12 h a high intensity blue light was used for complete polymerization of the methacrylate. 3. Placed in the heat of the oven for 24 h to finish the polymerization of the benzoyl peroxide.

Once polymerized, the block was removed from the mold. The next step was to make a preliminary cut to approximate the area of interest closer to the surface of the block. This was done with the help of a band saw (Exakt 300 CP) and irrigation to avoid overheating the sample, which would damage the tissues surrounding the implant. Then, to preserve the parallelism of the cuts to be made, the blocks were mounted on an acrylic sheet with the help of a resin (Technovit 4000^®^-Heraus Kulzer GMBH, Werheim, Germany) using a gluing press and a vacuum pump (Exakt 401), which holds the holder to the top of the press. The resin was spread on the back of the block, so that the part to be examined contacts the part underneath the gluing press. When polymerization was complete, the block was ready to be polished. The surface was polished using 1200 grit paper.

Sections were then stained with toluidine blue (Toluidine Blue O, Fisher Scientific, Hampton, NH, USA) for 20 min. The histopathologic and histometric analyses were performed with a digital camera system (DP12, Olympus, Japan) attached to a light microscope (BX51, Olympus, Japan) and an image analyzer software (MicroImage 4.0, Olympus, Japan).

### 2.10. Statistical Analysis

Data were recorded using a Microsoft Excel spreadsheet (Microsoft^®^, Redmond, Washington DC, WA, USA) and subsequently processed with the Stata 14 package (StataCorp^®^, College Station, San Antonio, TX, USA). Means and standard deviations were calculated, except for the granulometry test, where the mode and percentiles were used.

## 3. Results and Discussion

**Figure 3:**
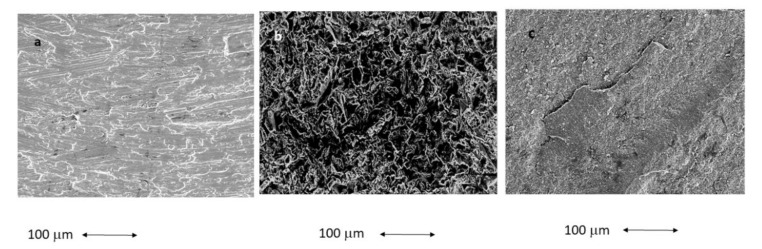
Scanning electron microscopy images of the surfaces of the different dental implants. (**a**) Ti, (**b**) Ti-R, and (**c**) ZrO_2_.

From Figure 3 the different topographies of the different dental implants can be observed. In Table 1 the different chemical compositions of the dental implants are shown, the metallic implants were obtained by EDS microanalysis and for the zircona the X-Ray diffractometer was used in order to obtain the percentages of the oxides using Rieveld analysis, as is usual for ceramic materials.

The roughness measurements can be observed in Table 2. R_a_ and R_z_, reveal that the results do not present statistical significance difference between Ti and ZrO_2_. This allowed us to determine the influence of the nature of the dental implant between titanium and zirconia. It can be seen that titanium treated by sand blasting with alumina has a roughness with statistically significant differences with respect to non-rough dental implants, which allowed us to determine the influence of roughness on the cyclic mechanical behavior and osseointegration. The ZrO_2_ dental implants could not be sandblasted because the lack of toughness of the ceramic caused cracks on the surface and the roughness obtained was very low. This fact did not allow us to achieve rough zirconia dental implants [24,25,26].

The CA and SFE calculations are shown in (Table 3). The sandblasting treatment decreased the wettability, and, therefore, increased the CA (increase of the hydrophobic character). ZrO_2_ implants presented the lowest values of contact angle, but this result does not present statistical significance difference with the Ti (*p* < 0.001). The Ti-R implants present lower total surface energy (SFE) than the smooth ones, especially the dispersive component, the results were similar between the Ti and ZrO_2_ without statistical significance differences (*p* < 0.001). However, the roughness produces a higher polar component than with the smooth dental implants, with significant differences between Ti-R and Ti and ZrO_2_ (*p* < 0.001).

The similar wettability and surface energy results between titanium and zirconia are due to the fact that the titanium surface is coated with the naturally occurring TiO_2_ layer and stabilized by the passivation treatment. Therefore, the liquid phase interaction occurs with transition metal oxides with very similar characteristics [51,52,53,54].

It should be noted that the increase in hydrophobic capacity and the decrease in surface energy, especially the dispersive component with respect to the polar component, favor the selective adsorption of proteins, especially fibronectin, which acts in a very important way as one of the precursor proteins of osteoblastic cell migration [52,53,54,55,56,57]. Therefore the physicochemical properties of the rough surface will favor osseointegration [57,58].

Table 4 confirms the compressive character of the residual stresses. As expected, the compressive stresses induced by sand blasting are statistically significant (*p* < 0.001, *t*-Student) and highly different from those induced on Ti and ZrO_2_. The results of Ti and ZrO_2_ present statistical significance differences (*p* < 0.001). The surface with the highest residual stress is the Ti-R surface, as the projection of abrasive particles at a pressure of 2.5 bar causes a compressive stress state on the surface. The residual stresses of Ti are due to the machining processes and those of ZrO_2_ to the sintering process, but the high temperatures cause the release of stresses, obtaining surfaces with very low stress state values [59,60].

**Figure 4:**
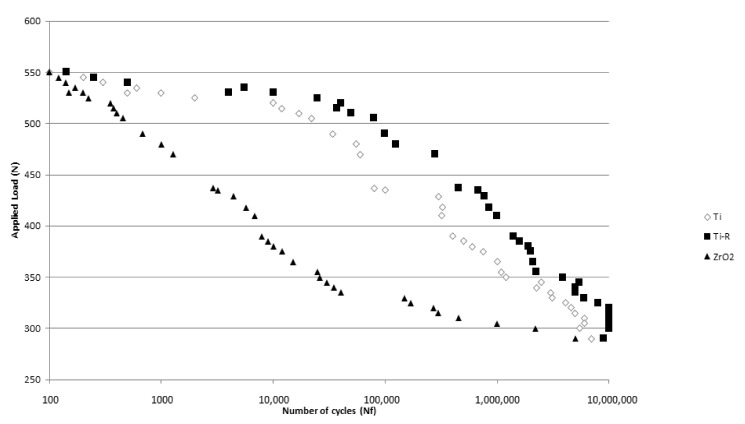
S–N curves for the different dental implants studied.

Figure 4 shows the number of cycles to failure (N_f_) at different loads applied for each type of implant. It can be observed that the Ti-R implants present longer life fatigue than the Ti and ZrO_2_. The fatigue behavior of the samples submitted to sand blasting treatment is better due to the compressive effect of the residual stresses on the surface which makes the crack nucleation difficult.

These results should be considered by clinicians as ZrO_2_ implants withstand little cycling at high loads. It is for this reason that this type of implant should be discouraged for patients with bruxism or high masticatory loads. Ti-R dental implants offer highly reliable results throughout the life of the dental implants [26,27].

**Figure 5:**
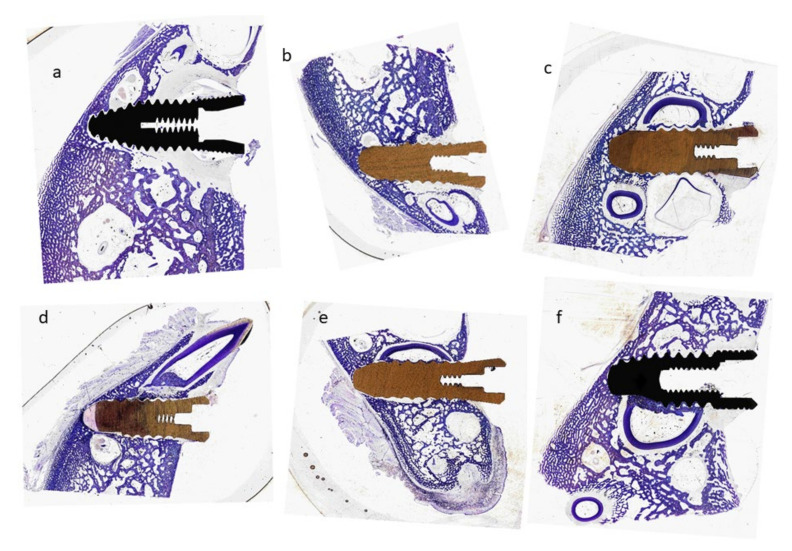
Histologies of the dental implants (Ti, ZrO_2_ and Ti-R) inserted in the tibiae of minipigs for 4 and 12 weeks. (**a**) Ti dental implant for 4 weeks. (**b**) ZrO_2_ dental implant for 4 weeks. (**c**) Ti-R dental implant for 4 weeks. (**d**) Ti dental implant for 12 weeks. (**e**) ZrO_2_ dental implant for 12 weeks. (**f**) Ti-R dental implant for 12 weeks.

Of the 90 dental implants placed, none had to be removed due to lack of fixation. All of them were of different degrees of osseointegration and with normal levels of inflammation, in no case was infection observed. Figure 5 shows the histology of the different types of dental implants for implantation times of 4 and 12 weeks.

The histology values shown in Figure 5 for each type of dental implant and the two implantation times show the formation of well-differentiated new bone tissue and good bone density especially in the area surrounding the dental implant. No giant cells or particle detachment from the dental implant are observed for either titanium or zirconia.

**Figure 6:**
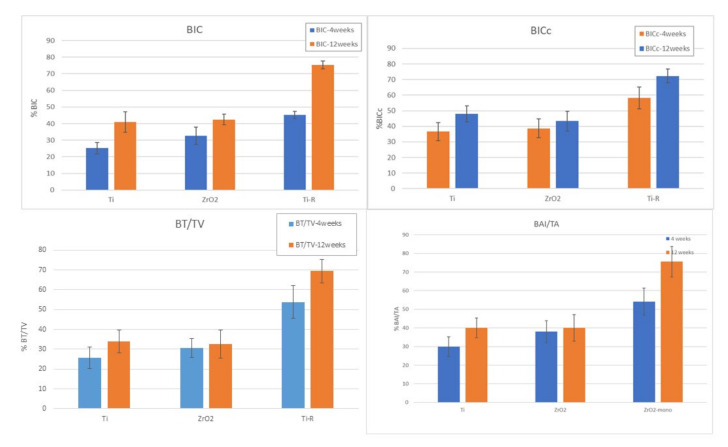
BIC, BICc, BT/TV, BAI/TA results for each dental implant and for different times of implantation.

Figure 6 shows the bone index contact values; from which it can be determined that the bone contact values for 4 weeks range from 25% for Ti dental implants to 42% for Ti-R implants. ZrO_2_ presents 31% of BIC. The difference is statistically significant with a *p* < 0.05 between the Ti and ZrO_2_ in relation to the Ti-R, the differences are not statistically significant between Ti and ZrO_2_. The BIC results at 12 weeks after implantation show a very similar bone index contact for the Ti (41%) and ZrO_2_ (42%) dental implants compared to the results for the zirconia implants. However, the Ti-R increases in 12 weeks with values higher than 70%.

As expected, the BICc values, shown in Figure 6, follow the same trend as the BIC. It can be seen that in this case the BICc in the case of titanium bone level is higher than zirconia dental implants, although there is no statistically significant difference at *p* < 0.05. The results of the percentage of new bone tissue BV/TV, the bone growth between the threads fillets and the amount of bone generated in relation to the total surface area show the high capacity to form new bone tissue of the Ti-R implants. It should be noted that the threads of the dental implants form a good amount of neoformed bone and therefore in all three cases a good mechanical anchorage can be guaranteed. The same behavior is observed in the new bone formed in relation to the total bone (BT/TV) for each dental implant and for different times of implantation [58,59,60,61].

It can be seen from the osseointegration results that there is no significant difference in the nature of the manufacturing material between titanium and zirconia when the dental implants have the same design and the same roughness. In principle, they are two inert biomaterials with excellent biocompatibility and similar physical and chemical characteristics and the same level of osseointegration can be justified [57]. From these results it is also revealed that the roughness obtained by sandblasting treatments with alumina causes changes in the physico-chemical properties on the surface and also in the compressive stress state of its surface which makes the dental implant have a much higher bone contact surface than dental implants without roughness. Therefore, roughness is a key factor in the development of dental implants [25,49].

The osseointegration values of the rough dental implants studied in this work are within the BIC ranges between 48 and 80% for implants with the same connection and roughness [43,44,45,46,47,48,49,50,53,55,56,57,62,63]. In our case, they present values in the upper part of the range. It can be observed that for roughness values from 0.9 to 2 μm, a tendency can be seen that the increase in roughness causes a higher BIC index [46,47,48,49,50].

The values for zirconia implants show a more important variation with designs ranging from 25 to 50% [24,25,26,27,28,29]. In this case, as there is no roughness parameter, the design plays a fundamental role in osseointegration. As already mentioned, this study using the same designs for the three types of dental implants allows us to determine the importance of the implant material and, in the case of titanium, the roughness.

Electro-polished or shot-blasted dental implants with titanium oxide (low abrasiveness) show very similar osseointegration values and always much lower than rough implants. Dental implants made of zirconia have similar osseointegration results to those of polished titanium. However, dental implants made of monoblock zirconia in one piece, i.e., a single implant-abutment body, have slightly better osseointegration values [64,65]. These differences have no statistically significant differences *p* < 0.005 and are always much lower than the bone formation levels of rough dental implants [66].

Despite these results, zirconia dental implants have a higher degree of dental aesthetics that is recommended in some cases. In any case, clinicians using zirconia dental implants must also assess the aspects of bone formation and long-term mechanical reliability. In most cases, however, titanium dental implants with a good prosthetic solution give an excellent result with the security of mechanical and biological fixation as well as the reliability of many years of mechanical cycles.

The limitations of this study are that we only studied one design, it would be interesting to perform the same studies with different dental implant designs to verify that the results are similar to the implants in this study. Likewise, it would be of value to carry out the influence of the prosthesis to see if it modifies the experimental results in any way. We believe that this study confirms the role of roughness as a very important factor for osseointegration, leaving the nature of the dental implant material (titanium/zirconia) as a minor influence.

## 4. Conclusions

The study carried out with three types of dental implants (Ti, Ti-R and ZrO_2_) with the same design allowed us to demonstrate that for similar roughness of Ti and ZrO_2_ implants the wettability and physical-chemical properties were very similar due to the nature of the titanium and zirconia oxides. This fact produces very similar BIC values at 4 and 12 weeks, reaching values of around 45% at 12 weeks. ZrO_2_ dental implants offer the lowest fatigue resistance due to the brittle nature of the bioceramics. Ti-R dental implants show higher fatigue strength values than Ti implants due to the compressive residual stress on their surface which delays surface crack nucleation. Regarding the osseointegration capacity, the values are much higher than Ti and ZrO_2_ implants due to the roughness that increases the contact surface with the bone, but also because of the more suitable physico-chemical properties of their surfaces. These conclusions avoided the possible influence of the design of the dental implant and allowed us to see the effect of the variables of the nature of the dental implant material and also the effect of the roughness.

## Figures and Tables

**Table 1:** Dental chemical composition. Percentages in weight.

Implants	O	C	N	H	Al	Fe	Ti
Ti	0.35 ± 0.09	0.08 ± 0.02	0.05 ± 0.01	0.02 ± 0.01	0.12 ± 0.04	0.30 ± 0.09	Balance
Ti-R	0.92 ± 0.13	0.08 ± 0.02	0.03 ± 0.01	0.03 ± 0.01	2.56 ± 0.76	0.32 ± 0.08	Balance
Oxides				Percentage			
ZrO_2_(+HfO_2_)				95.5 ± 1.5			
Y_2_O_3_				4.0 ± 0.6			
Al_2_O_3_				0.5 ± 0.1			

**Table 2:** Roughness for each dental implant studied.

Implant	R_a_ (μm)	R_z_ (μm)
Ti	0.33 ± 0.18	3.10 ± 0.69
Ti-R	1.98 ± 0.39 *	9.98 ± 1.34 *
ZrO_2_	0.32 ± 0.19	3.00 ± 0.34

* indicates statistical differences significance.

**Table 3:** Values (mean ± standard deviation) of contact angle of water (WA) and diiodomethane (DIIO), and the estimated surface energy (ϒ) with their polar (ϒ^P^) and dispersive (ϒ^D^) components, for each surface treatment.

Sample	CA (°)		SFE (mJ/m^2^)		
	WA	DIIO	ϒ	ϒ^D^	ϒ^P^
Ti	75.66 ± 2.67	43.60 ± 1.72	38.16 ± 1.16	35.60 ± 0.82	2.56 ± 0.75
Ti-R	94.65 ± 2.87 *	47.67 ± 1.73 *	31.47 ± 0.90 *	21.53 ± 0.81 *	4.94 ± 0.47 *
ZrO_2_	70.08 ± 3,57	45.10 ± 1.89	40.69 ± 0.87	37.45 ± 0.98	3.24 ± 0.41

* indicates statistical differences significance.

**Table 4:** Surface residual stresses calculated at the four different types of Ti dental implant surfaces.

Implant	Residual Stress (MPa)
Ti	−250.2 ± 8.9 *
Ti-R	−440.9 ± 19.3 **
ZrO_2_	−190.3 ± 5.2 ***

* indicates statistical significance differences. *, ** and *** indicate statistical significance differences between the symbols.

## Data Availability

The authors can provide details of the research with requests by letter and comments on their needs.

## References

[1] Kim T.I., Han J.H., Lee I.S., Lee K.H., Shin M.C., Choi B.B. (1997). New titanium alloys for biomaterials: A study of mechanical and corrosion properties and cytotoxicity. Bio-Med Mater. Eng..

[2] Niinomi M., Nakai M. (2011). Titanium-Based Biomaterials for Preventing Stress Shielding between Implant Devices and Bone. Int. J. Biomater..

[3] Chen L.-Y., Cui Y.-W., Zhang L.-C. (2020). Recent Development in Beta Titanium Alloys for Biomedical Applications. Metals.

[4] Niespodziana K., Jurczyk K., Jurczyk M. (2008). The synthesis of titanium alloys for biomedical applications. Rev. Adv. Mater. Sci..

[5] Uporabo B. (2017). A review of the surface modifications of titanium alloys for biomedical applications. Mater. Tehnol..

[6] Nicula R., Lüthen F., Stir M., Nebe B., Burkel E. (2007). Spark plasma sintering synthesis of porous nanocrystalline titanium alloys for biomedical applications. Biomol. Eng..

[7] Bannon B.P., Mild E.E., Luckey H.A., Kubli F. (1981). Titanium Alloys for Biomaterial Application: An Overview. Titanium Alloys in Surgical Implants. ASTM-STP796.

[8] Lemons J.E., Ratner B.D., Hoffman A.S., Schoen F.J., Lemons J.E. (1996). Application of Materials in Medicine and Dentistry. Dental Implants. Biomaterials Science: An Introduction to Materials in Medicine.

[9] Velasco-Ortega E., Alfonso-Rodríguez C.A., Monsalve-Guil L., España-López A., Jiménez-Guerra A., Garzón I., Alaminos M., Gil F.J. (2016). Relevant aspects in the surface properties in titanium dental implants for the cellular viability. Mater. Sci. Eng. C.

[10] Herrero-Climent M., Lázaro P., Rios J.V., Lluch S., Marqués M., Guillem-Martí J., Gil F.J. (2013). Influence of acid-etching after grit-blasted on osseointegration of titanium dental implants: In vitro and in vivo studies. J. Mater. Sci. Mater. Med..

[11] Gil F.J., Rodríguez D., Planell J.A., Cortada M., Giner L., Costa S. (2000). Galvanic corrosion behaviour of Titanium implants coupled to dental alloys. J. Mater. Sci. Mater. Med..

[12] Gil F.J., Sánchez L.A., Espias A., Planell J.A. (1999). In vitro corrosion behaviour and metallic ion release of different prosthodontic alloys. Int. Dent. J..

[13] Al-Hity R.R., Kappert H.F., Viennot S., Dalard F., Grosgogeat B. (2007). Corrosion resistance measurements of dental alloys, are they correlated?. Dent. Mater..

[14] Aparicio C., Gil F.J., Fonseca C., Barbosa M., Planell J.A. (2003). Corrosion behaviour of commercially pure tianium shot blasted with different materials and sizes of shot particles for dental implant applications. Biomaterials.

[15] Rodrigues D., Valderrama P., Wilson T., Palmer K., Thomas A., Sridhar S., Sadhwani C. (2013). Titanium Corrosion Mechanisms in the Oral Environment: A Retrieval Study. Materials.

[16] Variola F., Lauria A., Nanci A., Rosei F. (2009). Influence of Treatment Conditions on the Chemical Oxidative Activity of H2SO4/H2O2Mixtures for Modulating the Topography of Titanium. Adv. Eng. Mater..

[17] Variola F., Francis-Zalzal S., Leduc A., Barbeau J., Nanci A. (2014). Oxidative nanopatterning of titanium generates mesoporous surfaces with antimicrobial properties. Int. J. Nanomed..

[18] Cruz N., Gil J., Punset M., Manero J.M., Tondela J.P., Verdeguer P., Aparicio C., Rúperez E. (2022). Relevant Aspects of Piranha Passivation in Ti6Al4V Alloy DentalMeshes. Coatings.

[19] Chevalier J. (2006). What future for zirconia as a biomaterial?. Biomaterials.

[20] Camposilvan E., Leone R., Gremillarda L., Sorrentino R., Zarone F., Ferrari M., Chevalier J. (2018). Aging resistance, mechanical properties and translucency of different yttria-stabilized zirconia ceramics for monolithic dental crown applications. Dent. Mater..

[21] Piconi C., Maccauro G. (1999). Zirconia as a ceramic biomaterial. Biomaterials.

[22] Mohan P., Yuan B., Patterson T., Desai V.H., Sohn Y.H. (2007). Degradation of yttria-stabilized zirconia thermal barrier coatings by vanadium pentoxide, phosphorous pentoxide, and sodium sulfate. J. Am. Ceram. Soc..

[23] Lin J.-D., Duh J.-G. (2002). Fracture toughness and hardness of ceria- and yttria-doped tetragonal zirconia ceramics. Mat. Chem. Phys..

[24] Sevilla P., Sandino C., Arciniegas M., Martínez-Gomis J., Peraire M., Gil F.J. (2010). Evaluating mechanical properties and degradation of YTZP dental implants. Mater. Sci. Eng. C.

[25] Wenz H.J., Bartsch J., Wolfart S., Kern M. (2008). Osseointegration and clinical success of zirconia dental implants: A systematic review. Int. J. Prosthodont..

[26] Yılmaz E., Feyza N., Gökçe A., Fındık F. (2020). Production and Characterization of a Bone-Like Porous Ti/Ti-Hydroxyapatite Functionally Graded Material. J. Mater. Eng. Perform..

[27] Yılmaz E., Gökçe A., Findik F., Gulsoy O. (2018). Assessment of Ti–16Nb–xZr alloys produced via PIM for implant applications. J. Therm. Anal. Calorim..

[28] Pereira G.K.R., Guilardi L.F., Dapieve K.S., Kleverlaan C.J., Rippe M., Valandro L.F. (2018). Mechanical reliability, fatigue strength and survival analysis of new polycrystalline translucent zirconia ceramics for monolithic restorations. J. Mech. Beh. Biomed. Mater..

[29] Gil J., Delgado-García-Menocal J.A., Velasco-Ortega E., Bosch B., Delgado L., Pérez-Antoñanzas R., Fernández-Fairén M. (2021). Comparison of zirconia degradation in dental implants and femoral balls: An X-ray diffraction and nanoindentation study. Int. J. Implant. Dent..

[30] Gottlow J., Barkarmo S., Sennerby L. (2012). An experimental comparison of two different clinically used implant designs and surfaces. Clin. Implant Dent. Relat. Res..

[31] Scarano A., Degidi M., Iezzi G., Petrone G., Piattelli A. (2006). Correlation between implant stability quotient and bone-implant contact: A retrospective histological and histomorphometrical study of seven titanium implants retrieved from humans. Clin. Implant Dent. Relat. Res..

[32] Karl M., Irastorza-Landa A. (2017). Does implant design affect primary stability in extraction sites?. Quintessence Int..

[33] Irinakis T., Wiebe C. (2009). Initial torque stability of a new bone condensing dental implant. A cohort study of 140 consecutively placed implants. J. Oral Implantol..

[34] O’Sullivan D., Sennerby L., Meredith N. (2004). Influence of implant taper on the primary and secondary stability of osseointegrated titanium implants. Clin. Oral Implants. Res..

[35] Kim Y.K., Lee J.T., Lee J.-Y., Yi Y.-J. (2013). A randomized controlled clinical trial of two types of tapered implants on immediate loading in the posterior maxilla and mandible. Int. J. Oral Maxillofac. Implant..

[36] Javed F., Ahmed H.B., Crespi R., Romanos G.E. (2013). Role of primary stability for successful osseointegration of dental implants: Factors of influence and evaluation. Interv. Med. Appl. Sci..

[37] Trisi P., Berardini M., Falco A., Vulpiani M.P. (2015). Effect of Implant Thread Geometry on Secondary Stability, Bone Density, and Bone-to-Implant Contact: A Biomechanical and Histological Analysis. Implant. Dent..

[38] Lan T.H., Du J.K., Pan C.Y., Lee H.E., Chung W.H. (2012). Biomechanical analysis of alveolar bone stress around implants with different thread designs and pitches in the mandibular molar area. Clin. Oral. Investig..

[39] Ryu H.-S., Namgung C., Lee J.-H., Lim Y.-J. (2014). The influence of thread geometry on implant osseointegration under immediate loading: A literature review. J. Adv. Prosthodont..

[40] Wilson T.G., Miller R.J., Trushkowsky R., Dard M. (2016). Tapered Implants in Dentistry: Revitalizing Concepts with Technology: A Review. Adv. Dent. Res..

[41] Marković A., Calvo-Guirado J.L., Lazić Z., Gómez-Moreno G., Ćalasan D., Guardia J., Čolic S., Aguilar-Salvatierra A., Gačić B., Delgado-Ruiz R. (2013). Evaluation of primary stability of self-tapping and non-self-tapping dental implants. A 12-week clinical study. Clin. Implant. Dent. Relat. Res..

[42] Deligianni D.D., Katsala N.D., Koutsoukos P.G., Missirlis Y.F. (2000). Effect of surface roughness of hydroxyapatite on human bone marrow cell adhesion, proliferation, differentiation and detachment strength. Biomaterials.

[43] Aparicio C., Rodriguez D., Gil F.J. (2011). Variation of roughness and adhesion strength of deposited apatite layers on titanium dental implants. Mater. Sci. Eng. C.

[44] Von Wilmowsky C., Moest T., Nkenke E., Stelzle F., Schlegel K.A. (2014). Implants in bone: Part I. A current overview about tissue response, surface modifications and future perspectives. Oral Maxillofac. Surg..

[45] Albrektsson T., Wennerberg A. (2004). Oral implant surfaces: Part 1-review focusing on topographic and chemical properties of different surfaces and in vivo responses to them. Int. J. Prosthodont..

[46] Albrektsson T., Branemark P.I., Hansson H.A., Lindstrom J. (1981). Osseointegrated titanium implants. Requirements for ensuring a long-lasting, direct bone-to-implant anchorage in man. Acta Orthop. Scand..

[47] Fukuda A., Takemoto M., Saito T., Fujibayashi S., Neo M., Pattanayak D.K., Sasaki K., Nishida N., Kokubo T., Nakamura T. (2011). Osteoinduction of porous Ti implants with a channel structure fabricated by selective laser melting. Acta Biomater..

[48] Velasco-Ortega E., Monsalve-Guil L., Jiménez-Guerra A., Ortiz I., Moreno-Muñoz J., Nuñez-Marquez E., Pequeroles M., Perez R.A., Gil F.J. (2016). Importance of the roughness and residual stresses of dental implants on fatigue and osseointegration behavior. In vivo study in rabbits. J. Oral Implantol..

[49] Nicolas-Silvente A.I., Velasco-Ortega E., Ortiz-Garcia I., Monsalve-Guil L., Gil J., Jimenez-Guerra A. (2020). Influence of the Titanium Implant Surface Treatment on the Surface Roughness and Chemical Composition. Materials.

[50] Nkenke E.L.B., Weinzierl K., Thams U., Neugebauer J., Steveling H., Radespiel-Troger M., Neukam F.W. (2003). Bone contact, growth, and density around immediately loaded implants in the mandible of mini pigs. Clin. Oral Implant. Res..

[51] Kuchler U., Pfingstner G., Busenlechner D., Dobsak T., Reich K., Heimel P., Gruber R. (2012). Osteocyte lacunar density and area in newly formed bone of the augmented sinus. Clin. Oral Implant. Res..

[52] Schlegel K.A., Donath K., Rupprecht S., Falk S., Zimmermann R., Felszeghy E., Wiltfang J. (2004). De novo bone formation using bovine collagen and platelet-rich plasma. Biomaterials.

[53] Osman R.B., Swain M.V. (2015). A critical review of dental implant materials with an emphasis on Titanium versus zirconia. Materials.

[54] Hisbergues M., Vendeville S., Vendeville P. (2009). Review: Zirconia: Established facts and perspectives for a Biomedical in dental implantology. J. Biomed. Mater. Res. Part B Appl. Biomater..

[55] Osman R.B., Swain M.V., Atieh M., Ma S., Duncan W. (2014). Ceramic implants (Y-TZP). Are they a viable alternative to titanium implants for the support of overdentures? A randomized clinical trial. Clin. Oral Implant. Res..

[56] Han C.H., Johansson C.B., Wennerberg A., Albrektsson T. (1998). Quantitative and qualitative investigations of surface enlarged titanium and titanium alloys implants. Clin. Oral Implant. Res..

[57] Martin J.Y., Schwartz Z., Hummert T.W., Schraub D.M., Simpswon J., Lankford J., Dean D.D., Cochran D.L., Boyan B.D. (1995). Effect of titanium surface roughness on proliferation, differentiation and protein synthesis of human osteoblast-like vcells (MG63). J. Biomed. Mater. Res..

[58] Boyan B.D., Hummert T.W., Dean D.D., Schwartz Z. (1996). Role of material surfaces in regulating bone and cartilage cell response. Biomaterials.

[59] Manero J.M., Gil F.J., Padros E., Planell J.A. (2003). Applications of environmental scanning electron microscopy (ESEM) in biomaterials field. Microsc. Res. Tech..

[60] Kasemo B., Gold J. (1999). Implant Surfaces and Interface Processes. Adv. Dent. Res..

[61] Ronold H.J., Lyngstadaas S.P., Ellingsen J.E. (2003). Analysing the optimal value for titanium implant roughness in bone attachment using a tensile test. Biomaterials.

[62] Buser D., Schenk R.K., Steinemann S., Fiorellini J.P., Fox C.H. (1991). Influence of surface characteristics on bone integration of titanium implants. A histomorphometric study in miniature pigs. J. Biomed. Mater. Res..

[63] Bosshardt D., Chappuis V., Buser D. (2017). Osseointegration of titanium, titanium alloy and zirconia dental implants: Current knowledge and open questions. Periodontology.

[64] Depprich R. (2008). Osseointegration of zirconia implants compared with titanium: An in vivo study. Head Face Med..

[65] Gahlert M., Röhling S., Wieland M., Sprecher C.M., Kniha H., Milz S. (2009). Osseointegration of zirconia and titanium dental implants: A histological and histomorphometrical study in the maxilla of pigs. Clin. Oral Implant. Res..

[66] Koch F.P., Weng D., Krämer S., Biesterfeld S., Jahn-Eimermacher A., Wagner W. (2010). *Osseointegration* one-piece zirconia implants compared with a titanium implant of identical design: A histomorphometric study in the dog. Clin. Oral Implant. Res..

